# Lysosome-associated membrane glycoprotein (LAMP) – preliminary study on a hidden antigen target for vaccination against schistosomiasis

**DOI:** 10.1038/srep15069

**Published:** 2015-10-16

**Authors:** Sujeevi S. K. Nawaratna, Geoffrey N. Gobert, Charlene Willis, Jason Mulvenna, Andreas Hofmann, Donald P. McManus, Malcolm K. Jones

**Affiliations:** 1 School of Veterinary Sciences, The University of Queensland, Gatton Campus, Gatton Qld, 4343, Australia; 2 QIMR Berghofer Medical Research Institute, 300 Herston Road, Herston, Qld, 4006, Australia; 3 Structural Chemistry Program, Eskitis Institute, Griffith University, Brisbane, Qld 4111, Australia; 4 Faculty of Veterinary and Agricultural Sciences, The University of Melbourne, Parkville, Victoria 3010, Australia

## Abstract

Our previously reported gene atlasing of schistosome tissues revealed transcripts that were highly enriched in the digestive tract of *Schistosoma mansoni*. From these, we selected two candidates, *Sm*-LAMP and *Sm*-NPC2 for testing as vaccine targets. The two molecules were selected on the basis of relatively high expression in the gastrodermis, their potentially important biological function, divergence from homologous molecules of the host and possible apical membrane expression in the gastrodermis. Bacterially expressed recombinant peptides corresponding to regions excluding trans-membrane domains of the selected vaccine targets were used in blinded vaccine trials in CBA mice using alum-CpG as adjuvant. Vaccine trials using the recombinant insoluble *Sm*-LAMP protein showed 16–25% significant reduction in total worm burden. Faecal egg count reduction was 52% and 60% in two trials, respectively, with similar results for the solubly expressed protein. Liver egg burden was reduced significantly (20% and 38%) with an insoluble recombinant *Sm*-LAMP in two trials, but not with the soluble recombinant form. Parasite fecundity was not affected by either *Sm*-LAMP protein preparations in the trials. It is concluded that *Sm*-LAMP may provide limited protection towards *S. mansoni* infections but could be used in combination with other vaccine candidates, to provide more comprehensive protection.

Schistosomiasis, a neglected tropical disease affecting over 200 million people in 76 countries, is caused by infection with parasitic flatworms of the genus *Schistosoma*^1^. Currently, the only effective treatment for all forms of schistosomiasis is the drug praziquantel. Continued use of this single drug may be problematic for a number of reasons, including the possibility of reduced sensitivity of schistosomes to the compound^2^,^3^,^4^,^5^, its lack of efficacy against immature schistosomes^6^ as well as the lack of protection against reinfection. In highly endemic regions, the success of praziquantel can be limited by constant reinfection. Therefore, there is an urgent need for alternative treatments, including new drugs and vaccines for countering the possibility of emerging drug resistance.

Over the years there have been many vaccine candidates tested against *Schistosoma* infections including irradiated whole parasites^7^,^8^,^9^, whole parasite extracts, recombinant peptides and DNA based vaccines^10^,^11^. In the 1990’s six antigens were selected by the WHO for further research^12^, but of these, only fatty acid binding protein (Sm14) and glutathione-*S*-transferase (Sm28 GST) have reached clinical trials^13^. Two *S. mansoni* tetraspanins (TSP 1 and TSP2), which are tegument-associated molecules originally uncovered by signal sequence trap methods and tegument proteomic surveys, have shown promising vaccine efficacies^13^,^14^. Another outer membrane protein of the tegument of *S. mansoni*, Sm29, is a promising vaccine target against which putative resistant individuals in endemic settings have developed a strong IgG1 and IgG3 isotype response^15^,^16^. A recent DNA vaccine against the large subunit of *S. mansoni* calpain (Sm-p80) has been tested in mice effectively against both *S. japonicum* and *S. mansoni*^11^. Prominent targets of vaccination have been the surface lining of the schistosome parasites, the tegument^13^, a primary site of host interaction.

Schistosomes possess a simple gastrovascular cavity for digestion. This blind digestive system consists of a mouth, an oesophageal region from which emerges the caecal region, which functions in digestion and nutrient absorption. The lining of the caecum is a syncytial epithelium, termed the gastrodermis. The schistosome digestive system becomes metabolically active soon after the parasite invades its mammalian host, differentiating and growing at a rate commensurate with the development of the parasite^17^,^18^,^19^. The primary role of the digestive system in blood-feeding schistosomes is to process large quantities of ingested blood. Adult female parasites are thought to process 330,000 erythrocytes per hour^20^. The schistosome gut is thus exposed to host blood and a variety of immune components soon after host invasion, possibly as early as two days after entering the liver^21^. Molecules expressed at the surface of the schistosome gastrodermis may provide good targets as vaccine candidates due to their location and importance in nutritional support of the parasite.

Molecules expressed at the surface of the schistosome gastrodermis are not naturally exposed to the host immune system in parasitism because they are secluded within the parasite. Some gastrodermal molecules are exposed to the host as vomitus or during disintegration after the worm’s death^22^. Consequently, the parasite is not under evolutionary pressure to develop mechanisms to evade the host immune response against these “hidden” molecules^23^. We reasoned that some molecules putatively bound at the surface of the gastrodermis may be targets for vaccination, in much the same way as hidden antigens of the sheep nematode, *Haemonchus contortus*^24^,^25^ and the cattle tick *Rhipicephalus microplus*^26^ have been targeted.

Here, we outline a pipeline of antigen discovery and testing in vaccination experiments. Although our study is not the first to test schistosome gut molecules as vaccine targets, our approach incorporates the identification of molecules enriched in the gastrodermis, a rigorous approach to antigen selection leading to functional characterisation and testing of candidate molecules as targets of immune prophylaxis. We focus on two antigens, a schistosome lysosome-associated membrane glycoprotein (*Sm*-LAMP) and Niemann Pick type C2 protein (*Sm*-NPC2). This approach represents a new alternative for *Schistosoma* vaccine discovery.

## Results

### Selection of antigens

**Figure 1:**
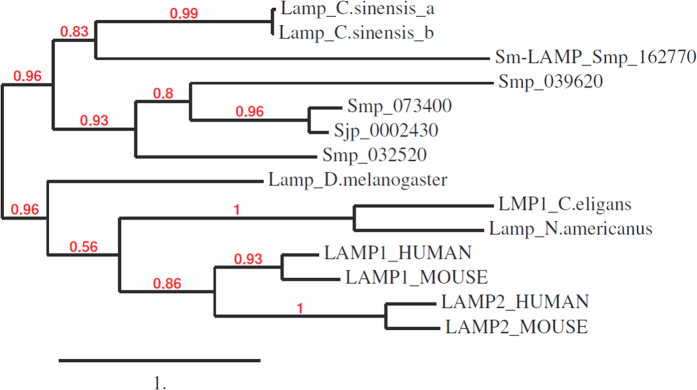
Comparison of *S. mansoni* LAMP with human and mouse LAMP1 and LAMP2 using ClustalW. *Sm*-LAMP was compared with mouse and human orthologues and other schistosome LAMP proteins. The tree shows that the schistosome molecules clearly bifurcate from mammalian counterparts.

We previously reported that 393 genes were up-regulated in the gastrodermis of adult female worms of *S. mansoni* compared with whole female parasite tissue^27^. Blast2GO analysis provided specific annotations and descriptions for 126 genes and gene products. The main characteristics used in the selection of genes for vaccine trials are summarised in Supplementary Table 1. Of the 126 annotated genes transcripts that were enriched in the gastrodermis of *S. mansoni*^28^, 23 had putative orthologues that were also up-regulated in the gastrodermis of *S. japonicum*^29^. The genes were then scrutinised for the following criteria: enrichment in the gastrodermis relative to other tissues, important biological function, motifs consistent with membrane association and sufficient sequence divergence from human and mouse (host) orthologues (Fig. 1, Supplementary Fig. 1). Of these 23 genes, two targets were selected, namely a LAMP (enriched 2.7-fold in *S. mansoni*, 46-fold in *S. japonicum*) and a Niemann Pick type C2 protein (*Sm*-NPC2) (3.1-fold in *S. mansoni*, 120 fold in *S. japonicum*). ClustalW multiple sequence alignments were used to calculate the percentage identity of the two selected vaccine targets with homologous sequences from human and murine hosts. *Sm*-LAMP had only 13% and 15% sequence identity with human LAMP 1 and 2, respectively. Mouse LAMP 1 and 2 had sequence identities of 14% and 15% to *Sm*-LAMP, respectively. *Sm*-NPC2 sequence had 32% identity to human and 31% to mouse NPC2.

### Real-time PCR

**Figure 2:**
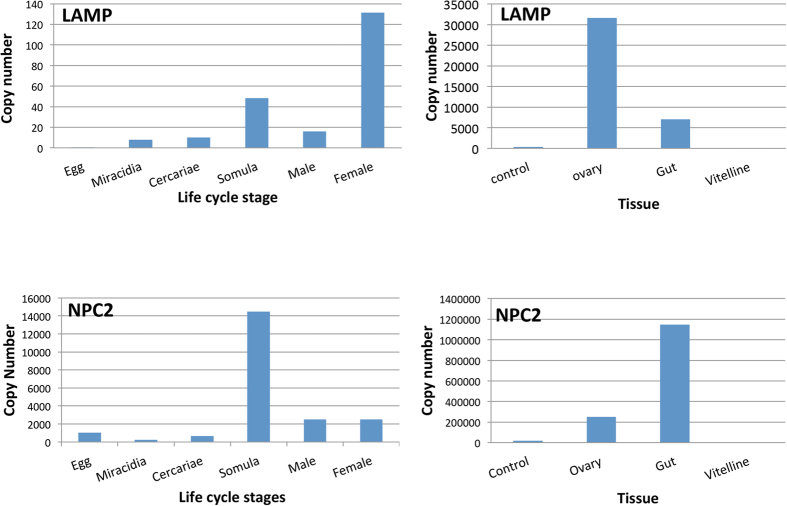
Differential expression of *Sm*-NPC2 and *Sm*-LAMP throughout the *S. mansoni* lifecycle stages and in different biologically important tissues of the *S. mansoni* adult female.

The relative transcript abundance of *Sm*-NPC2 and *Sm*-LAMP was investigated for a range of life cycle stages of *S. mansoni*, and in different biologically important tissues of the *S. mansoni* adult female (Fig. 2). Both *Sm*-NPC2 and *Sm*-LAMP were enriched in schistosomula and adult male and female worms, that is, in stages associated with human parasitism.

### Protein expression

#### Appraisal of Sm-LAMP recombinant protein structure

**Figure 3:**
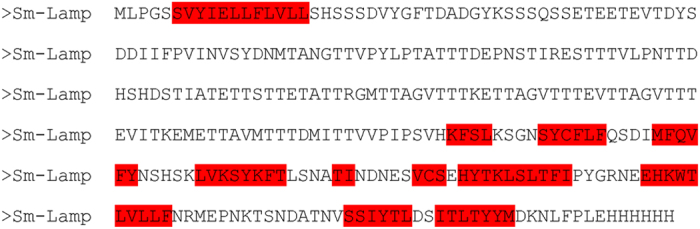
Sequence of *Sm*-LAMP recombinant protein with C terminal hexa-histidine tag. Areas marked in red show the predicted beta strands.

**Figure 4:**
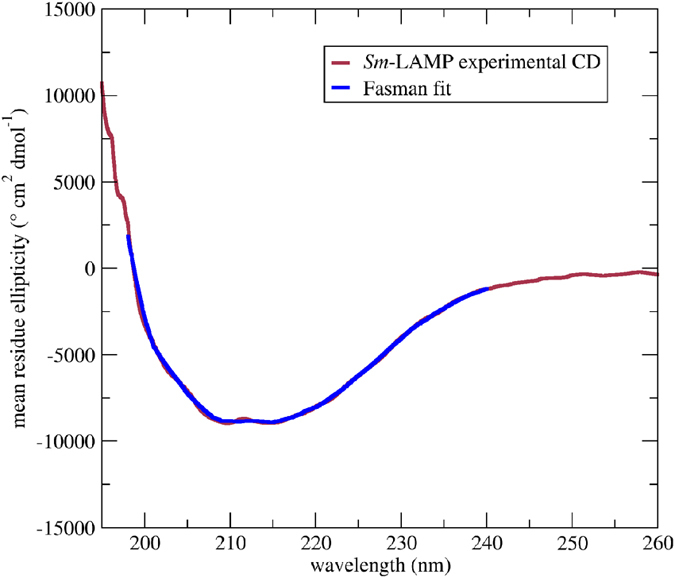
CD spectra analysis of soluble *Sm*-LAMP recombinant protein in 20 mM NaCl, 10 mM Na_2_HPO_4_ at pH 8.0. The experimental data (red) fit well with the predicated Fasman fit (blue).

The amino acid sequence of *Sm*-LAMP was analysed using the PSIPRED v3.0 secondary structure prediction algorithm^30^. The predicted structure suggested that the schistosome molecule consists of 24% beta-strands and 76% unstructured regions (Fig. 3). The CD spectrum of soluble *Sm*-LAMP showed excellent agreement with the expected secondary structure content, and fits using three Fasman prototype structural elements (helix, strand, random) yielded 8% alpha-helix, 16% beta-strand and 75% unstructured regions (Fig. 4).

#### Western blots

**Figure 5:**
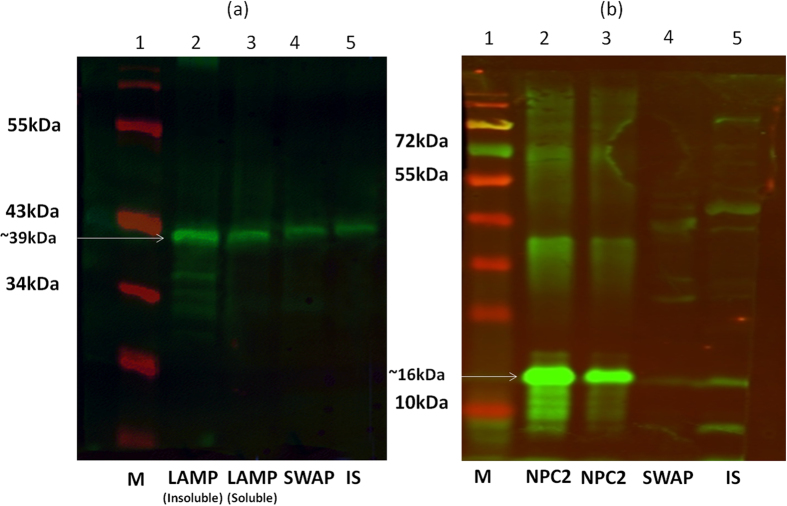
Western blots performed with the rabbit polyclonal antibodies. Rabbit plyclonal antibodies made against *Sm*-LAMP (**a**) and *Sm*-NPC2 (**b**) were used against the respective recombinant protein (lanes 2 and 3), SWAP (lane 4) and insoluble fraction of the crude worm protein extract (lane 5). Lane 1- molecular weight markers.

Rabbit polyclonal antisera in 1:500 dilutions were able to detect the recombinant proteins and native proteins in the soluble worm antigen preparation (SWAP) and insoluble protein (IS) fractions of worm homogenates. *Sm*-LAMP and *Sm*-NPC2 were detected in both SWAP and IS (Fig. 5). *Sm*-LAMP is present as a 64 kDa band in Western blot analysis which is double the size expected (33.6 kDa). Mass spectrometry fingerprinting after limited proteolysis performed on these gel bands confirmed the protein as Sm-LAMP.

#### Immunolocalisation

**Figure 6:**
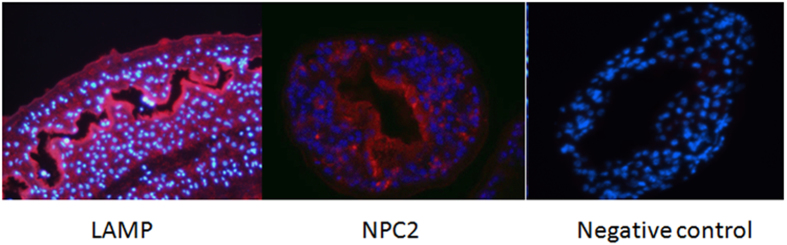
Immunofluorescence of *S. mansoni* female worm sections. Rabbit plyclonal antibodies made against *Sm*-LAMP and *Sm*-NPC2 antibodies were used in the two sections respectively. Rabbit pre-immune sera were used for the negative control.

Immunolocalisation studies showed that all three proteins were localised to the gastrodermis and tegument of adult male and female parasites (Fig. 6).

#### Endotoxin levels

Preparations of soluble *Sm*-LAMP presented endotoxin levels of 6.95 EU/ml, whereas preparations of insoluble *Sm*-LAMP and *Sm*-NPC2 contained endotoxin levels of 8.48 and 9.54 EU/ml, respectively.

#### Vaccine trials

**Figure 7:**
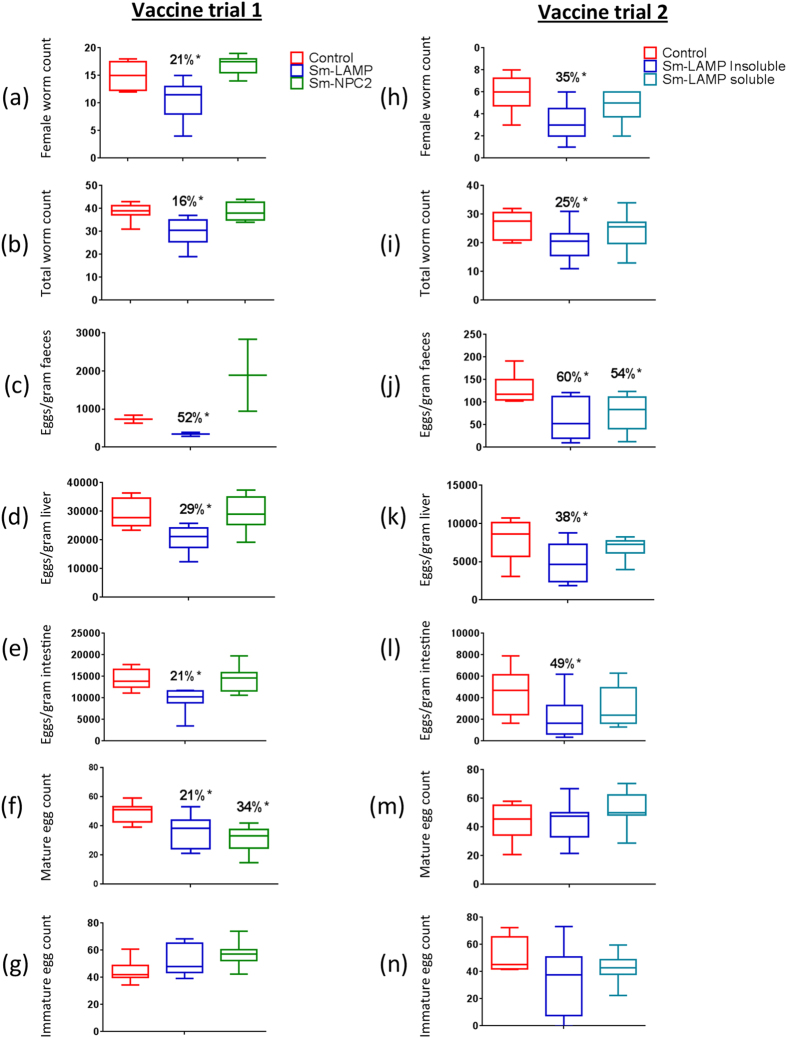
Comparison of worm counts and egg counts between the two vaccine trials. Percentage reduction is shown above each group where a significant reduction in numbers was observed. Error bars represent the standard error (n = 10). *p < 0.05. All graphs were created using GraphPad Prism 6.

The results of two vaccine trials (trial one with insoluble protein and trail two with soluble protein) are shown in Fig. 7 and Table 1. In the first vaccine trial, statistically significant reductions in egg and worm counts were obtained only with *Sm*-LAMP. The mean female worm count reduction was 20.93% (p < 0.05), whereas the total worm burden reduction was 16.21% (p < 0.05) (Fig. 7(a,b)). The mean percentage egg count reductions in faeces, liver and intestinal tissue were 51.9% (p = 0.001), 20% (p < 0.05) and 21% (p < 0.05), respectively (Fig. 7(c–e)). There was a significant reduction in the mature egg percentage in the intestinal wall for the *Sm*-LAMP (21%) and *Sm*-NPC2 (34%) vaccine groups with an increase in the percentage of immature eggs in the total egg population (Fig. 7(f,g)). However, *Sm*-NPC2 did not produce any significant protection in terms of reduced worm numbers nor reduced numbers of faecal, liver or intestinal eggs. The second vaccine trial resulted in significant worm burden and egg count reduction in the group immunised with the insoluble *Sm*-LAMP (Fig. 7(h,i)). Percentage egg reduction in liver, intestine and faeces (38%, 49% and 60%, respectively) showed reproducible results with that of the first vaccine trials for insoluble *Sm*-LAMP. Total and female worm counts also showed significant reduction (25% and 35.7%) when using the insoluble *Sm*-LAMP protein. However, the vaccine group immunised with soluble *Sm*-LAMP did not show any significant reduction in worm counts or egg counts. No significant reduction was observed in the percentage of mature eggs in oograms in either of the groups in the second vaccine trial with only 4% reduction in the group immunised with insoluble *Sm*-LAMP protein. Soluble *Sm*-LAMP showed a significant reduction in liver granuloma area (p = 0.003) when compared to the control group. There was no significant difference between the average lengths of male and female worms from vaccinated mice and the control mice in both vaccine trials. Table 1 shows a comparison of the results of the two vaccine trials for *Sm*-LAMP. The control groups of mice in both vaccine trials did not show any antibody response. Antibody response to IgG subtypes was greater for soluble antigen than the insoluble form except for IgG2a (Table 1).

## Discussion

LAMP is a major integral protein of the lysosomal membrane^31^, which plays an important role in the formation of phagosomes^32^. LAMP-2 has also been found to be involved in cholesterol transport^33^,^34^. LAMPs are characterised by abundant N-terminal glycosylation sites, a single transmembrane domain and a short cytosolic tail^35^,^36^. *Sm*-LAMP has four cystine residues of which one is conserved as compared to human, mouse, *S. japonicum* (Sjp_0002430) and other *S. mansoni* LAMP proteins (Smp_073400, Smp_032520, Smp_039620). The transmembrane region is also conserved when compared with human, mouse and other schistosome LAMPs (Supplementary Fig. 1). *Sm*-LAMP contains a lysosomal targeting signal (YXXØ, where X could be any amino acid and Ø is a bulky hydrophobic residue)^37^ in its cytosolic tail (YTTL) (Supplementary Fig 1). Interestingly, some members of the LAMP family have also been described as proteins expressed at the cell surface^38^,^39^. In our transcriptomic studies^27^ *Sm*-LAMP (Smp_162770) was shown enriched in the gastrodermis of females^29^. Other workers found the protein in the vomitus of adult worms^22^. These observations indicate that the molecule is abundant in the gut and potentially located at or near the surface of the gastrodermis^27^. Further, the presence of *Sm*-LAMP in the vomitus and cell membranes might explain why the protein appears in soluble adult worm antigen preparation (SWAP) and insoluble protein fractions of the worm.

NPC2 homologues are known as lysosomal molecules. We found *Sm*-NPC2 to be highly enriched in the gastrodermis of *S. mansoni* and *S. japonicum*^28^,^29^. *Sm*-NPC2 is also enriched in the *S. mansoni* ovary, an observation consistent with the detection of abundant NPC2 in the secretome of *S. mansoni* eggs^40^. NPC2 functions with NPC1 in the transport of cholesterol, glycolipids and other sterols in eukaryotic lysosomes^41^,^42^. In mice, NPC2 expression correlates with a cellular requirement for cholesterol, being abundant in tissues that use this sterol, such as the ovary and uterine cells, but not the oviduct^43^. A secretory form of NPC2 is also found in the gut lumen of mammals, where it may be involved in enhancing solubilisation of dietary cholesterol^44^,^45^. Similarly, *Sm*-NPC2 has been found in *S. mansoni* vomitus, implicating this molecule in surface expression and secretion by the gastrodermis^22^.

As discussed above, the two vaccine candidates we selected are membrane-resident molecules postulated to be related to cholesterol/lipid metabolism. Many eukaryote pathogens scavenge the majority of their nutrients including lipid and cholesterol^46^. Likewise, schistosomes lack the ability to synthesise sterols and lipids^47^ and, therefore, obtain these molecules from the host to meet their requirements^46^,^48^,^49^. Molecules related to cholesterol uptake and metabolism are likely to be important for schistsome survival within the mammalian host. As schistosomes are dependent on the human host for sterol and lipid requirements^47^, this is an additional advantage for the selected vaccine candidates in addition to being present as a hidden antigens.

Only *Sm*-LAMP reduced worm burdens, although the protection was moderate compared with other *Schistosoma* vaccine candidates^10^,^14^,^50^. Sterile immunity is not required from a schistosome vaccine^51^,^52^. Although there was significant worm count reduction in the mice immunised with *Sm*-LAMP, the number of eggs per female was not reduced. Therefore the egg count reductions were due to the reduction of the number of female worms, and not an effect on fecundity of individuals. In contrast, the oogram showed that the percentage of mature eggs reaching the intestine was reduced significantly, suggesting an effect on egg development. The higher reduction in faecal eggs, when compared to liver and intestinal egg counts, may also be due to the reduced number of mature eggs in the intestinal wall. The significant reduction of the liver granuloma area by *Sm*-LAMP shows that this vaccine candidate could result in a significant clinicopathological improvement of the host.

The soluble form of *Sm*-LAMP did not provide better vaccine efficacy but it resulted in a similar level of faecal egg reduction compared with the insoluble protein. Conversely, the ELISA results showed that the antibody response was slightly higher with the soluble protein. We tested mouse serum for the IgG response only. In humans, protein antigens commonly induce an IgG1 or IgG3 response (IgG2a and IgG2b mouse equivalent)^53^ but can also generate IgG4 (mouse IgG1) or IgE. Our results showed a better IgG2b and IgG1 response with the soluble protein antigen which conforms to the above pattern. This could be explained by the physico-chemical nature of the soluble antigen which may have better antigenic epitopes due to the protein adopting a native fold. However, the insoluble antigen showed a better response with the IgG2a subclass. Although ELISA analysis showed that the antibody response was better against the soluble protein, this correlation may not predict its complete protective effectiveness *in vivo*^54^. Therefore, antibodies raised against the soluble proteins could result in less protection (ie worm elimination) than those produced against insoluble protein. Future studies addressing the IgE response will be required to assess whether these may have contributed to the better protection observed with the insoluble antigen. Notably, there was a correlation between the IgE levels and the degree of protection against the *Schistosoma* infection in resistant individuals^55^ and a balanced Th1 and Th2 type response has been associated with better protection against the infection. Therefore the higher protection level observed with the insoluble protein might be due to a better balance in Th1/ Th2 response.

*Sm*-NPC2 was ineffective as a vaccine candidate. Although *Sm*-NPC2 was predicted to be present on the apical membrane of the gastrodermis and *in silico* analysis predicted good antigenicity, the true accessibility of antibodies specifically directed against these proteins may be poor in the gastrodermis. Accessibility is considered an obstacle in designing vaccines against outer tegument proteins because of the presence of the parasite’s membranocalyx^52^. Although the gastrodermis is not lined by a membranocalyx, other factors might have come into play to limit efficacy, among them limited accessibility of the molecule due to its location in the gastrodermal cytoplasm or the structure of the protein itself^52^. Also, the removal of transmembrane regions from the recombinant peptide may have had an effect on the final structure of the recombinant protein.

In conclusion, the use of proteins from the apical membranes of the gastrodermis is a possible avenue for selection of better vaccine candidates against schistosomiasis. Out of the two vaccine candidates selected in this study *Sm*-LAMP gave a reproducible moderate level of vaccine efficacy, whilst *Sm*-NPC2 cannot be recommended as a vaccine target. However, for the purpose of this study, *Sm*-NPC2 was a useful positive control, especially in mitigating any possible effect that may have arisen from bacterial endotoxin contaminations which were found to be similar for both proteins in the two vaccine trials. *Sm*-LAMP efficacy could be enhanced by investigating smaller peptides, using in combination with another moderate efficacious vaccine candidate or with praziquantel treatment. The presence of the antibodies to *Sm*-LAMP and *Sm*-NPC2 should be tested in putative resistant individuals (endemic normals) to confirm the true hidden nature of these targets. However, the absence of a response in the negative control group of mice in the vaccine trial supports our theory of the two vaccine targets being hidden from the host. Targeting antigens occurring upstream from the gastrodermis^56^,^57^,^58^, including the secreted components of the oesophageal gland region, may prove to be a better strategy. Although a relatively small tissue mass, the oesophageal gland appears as a highly metabolically active region in the initial processing of the host blood meal and, as such, contains many potential vaccine candidates. Molecules expressed in the oesophageal gland may then represent better hidden antigen targets since the antibodies generated there would be less exposed to digestive proteases of the worm, and additionally, this region develops earlier in the young worm. These antigens could be combined in the future for better hidden antigen vaccines. Therefore we conclude that *Sm*-LAMP provides marginal protection towards *S. mansoni* infections and has the potential to be used in combination with other vaccine candidates or with praziquantel treatment, to provide more comprehensive protection against schistosomasis.

## Materials and Methods

### Ethics Statement

Use of animals was approved by the Animal Ethics Committee of the QIMR Berghofer Medical Research Institute under project P1289. The study was conducted according to the guidelines of the National Health and Medical Research Council of Australia, as published in the Australian Code of Practice for the Care and Use of Animals for Scientific Purposes, 7th edition, 2004 (www.nhmrc.gov.au). All work related to live lifecycle stages was performed in quarantine-accredited premises.

### Selection of hidden antigens

Selection of antigens for this work followed extensive transcriptional profiling of individual tissues we previously reported for *Schistosoma mansoni*^27^. Genes enriched in the gastrodermis, with expression profiles 2 fold or higher, as compared to expression in the entire female worm, were further filtered *in silico* for the selection of potential targets as described below.

The annotations of all 393 up-regulated genes of the gastrodermal tissue were updated using Blast2GO software^59^. The annotation consisted of gene descriptions, gene ontologies and InterPro Scan results. Sequences were analysed for inferred membrane association (transmembrane domain), high expression in *S. mansoni* and *S. japonicum*^27^,^29^ gastrodermal tissues, and a biological function considered essential and potentially non-redundant. Additionally, the antigenicity and hydrophobicity of the peptides were predicted using MacVector™ 8.0 software (http://www.macvector.com) (Supplementary Fig. 2). From a panel of potential targets, two molecules, LAMP and a homologue of the Niemann Pick type C2 protein (NPC2) protein (Smp_194850) were chosen for further characterization and vaccine efficacy trials. The similarity of candidate molecules was assessed by comparing their amino acid sequences to those of human and mouse orthologues using ClustalW2 online alignment tool^60^. Sm LAMP is also compared to the three other LAMP proteins described in the Schistosoma Gene Database. A phylogenetic tree was drawn using Phylogeny.fr to compare *Sm*-LAMP with other schistosome, human and mouse orthologs.

### Real-time PCR

Relative abundance of the *Sm*-LAMP and *Sm*-NPC2 transcripts was determined for different *S. mansoni* life cycle stages (eggs, miracidia, cercariae, 5 day old schistosomula, adult males and adult females) and for microdissected digestive and reproductive tissues^27^ using quantitative real-time PCR (qPCR), to confirm that the selected genes are enriched in the gastrodermis and in life cycle stages within the mammalian host^27^. Forward and reverse primers (Sigma-Aldrich) were designed for *Sm*-LAMP and *Sm*-NPC2 using Primer3 (v.0.4.0) software (Supplementary Table 2). Total RNA samples were DNase treated (Promega) prior to synthesis of cDNA using a QuantiTect® Whole Transcriptome Kit (QIAGEN). All cDNA samples were diluted to 5 ng/μl. Real-time PCR was performed and analysed using previously described protocols^61^ using DNA segregation ATPase^62^ (Accession No. Smp176580) as the normalising housekeeping gene. Data were analysed using the Rotor Gene 6 software^63^.

### Protein expression 

#### Cloning

The predicted extra-cellular *Sm*-LAMP sequence consisting of 296 amino acids, with the removal of the less antigenic region adjacent to the transmembrane region (as predicted by MacVector™ 8.0 software) (Supplementary Fig. 2) was amplified by PCR from cDNA of *S. mansoni* adult female worms using the forward primer with a *Nco*1 restriction enzyme recognition site (in lowercase in the following sequence), catgccatggcgATGTTGCCAGGTAGCTCAGTTTATATTG and the reverse primer with a Xho1 enzyme recognition site (in lower case) ccgctcgagCGGAAATAAATTCTTATCCATATAATAAG with PfuTurbo® DNA polymerase (Agilent-Stratagene). The PCR product and the pET−28a (+) (Novagen) vector were digested (Nco1-HF and Xho1, New England BioLabs) and then dephosphorylated using CIP (FINNZYMES, Thermo Scientific). The products were purified by PCR using QIAquick® PCR Purification kit (QIAGEN), according to the manufacturer’s instructions, and then ligated (T4DNA Ligase (Promega) into the vector.

*Sm*-NPC2, cloned into pET100/D-TOPO® vector, was kindly provided by Dr. David L. Williams, Department of Immunology-Microbiology, Rush University, Chicago, Illinois.

The ligation mixture was added to One Shot® TOP 10 (Invitrogen) chemically competent *E. coli* cells and transformed by heat shock, followed by the addition of LB medium. Transformed cells were then plated onto LB agar plates containing 50 μg/ml Kanamycin sulphate and incubated overnight at 37 °C. LB agar plates containing 100 μg/ml Ampicillin were used for growing pET100/D-TOPO® vector with *Sm*-NPC2.

Ten single colonies per construct were screened for the presence of an insert using PCR with T7 (pET) promoter TAATACGACTCACTATAGGG and terminator CTAGTTATTGCTCAGCGGTG primers (Sigma-Aldrich). Colonies with confirmed inserts were grown overnight in 5 ml of LB medium with antibiotics. Plasmids were purified using the QIAprep® Spin Miniprep kit (QIAGEN) using a microcentrifuge. Clones were sequenced using Big Dye Terminator v3.1 (Amersham Bioscience, Australia) sequencing protocol to confirm the insert was in frame and did not contain any mutations.

#### Protein production and purification

For each protein, a plasmid containing the correct, in-frame sequence was transformed into BL 21-(DE3) cells (Invitrogen) via heat shock as described above.

Cells from an overnight culture were used to inoculate 1 L of 2YT medium with appropriate antibiotic. Cultures were incubated in a shaker incubator until an OD_600_ ≈ 0.6 was reached and induced with  mM IPTG. For inclusion body production, expression was carried out at 37 °C for 5 hours. After cell harvest, lysis and separation of debris, the inclusion bodies were solubilised using a buffer containing 6 M guanidine hydrochloride and 20 mM beta-mercaptoethanol (BME) at room temperature for one hour. Solubilised *Sm*-LAMP was then purified using HIS-Select® Nickel affinity Gel (Sigma). A buffer containing 6 M urea and BME with varying concentrations of imidazole was used for elution. An Amicon Ultra-15 centrifugal filter device with a 10 kDa cutoff was used to concentrate the eluted protein. Imidazole and urea were removed by dialysing the concentrated protein against 1×PBS using Snake Skin® pleated dialysis tubing with a 10 kDa cutoff (Thermo Fisher Scientific).

For *Sm*-LAMP, soluble protein was obtained after modification of the above procedure such that expression was carried out at 16 °C for 24–28 hours. After cell lysis and removal of debris by centrifugcation, soluble protein was purified from the cytosolic contents using affinity chromatography as above, but without urea and BME.

All purified recombinant proteins were identified by Western blot with anti-His antibodies, as well as mass spectrometry fingerprinting after limited proteolysis. Endotoxin contamination of recombinant proteins was measured using Pierce LAL (Limulus Amebocyte Lysate) Chromogenic Endotoxin Quantitation Kit (Thermo Fisher Scientific Inc., IL, USA) according to the manufacturer’s instructions. All proteins used for vaccinations were tested at similar concentrations.

#### Appraisal of Sm-LAMP recombinant protein structure

The secondary structure of solubly expressed *Sm*-LAMP was assessed by acquiring CD (circular dichroism) spectrum of 3 mM *Sm*-LAMP in 20 mM NaCl, 10 mM Na_2_HPO_4_, pH 8.0 acquired on a Jasco J-715 CD/ORD spectropolarimeter. The data were fitted with Fasman standard curves using the software program ACDP^64^ to determined secondary structure content. These results were compared to secondary structure contents predicted by PSIPRED v3.0^30^.

#### Rabbit polyclonal sera

Rabbit polyclonal antibodies were produced commercially at the Institute of Medical and Veterinary Sciences (IMVS), Veterinary Services Division, Gilles Plains South Australia 5086. Each rabbit received 1.2 mg of insoluble protein with Freund’s adjuvant, in four doses (0.3 mg per dose) at 0, 3, 6 and 9 weeks. All sera were aliquoted and stored at −80 °C until further use.

#### Western blot

Rabbit polyclonal antisera were used to detect the presence of *Sm*-LAMP and *Sm*-NPC2 in a *S. mansoni* soluble worm antigen preparation (SWAP) and in the insoluble fraction. Fifty nanograms of protein (50 ng recombinant protein, SWAP and insoluble protein) were run in a SDS- PAGE and transferred onto a PVDF membrane, which had been soaked in 100% methanol and rinsed with sterile milliQ water. The membrane was incubated with Odyssey® blocking buffer (LI-COR® Biosciences) for 1 hour at room temperature. Specific primary rabbit polyclonal antibodies were added at 1:500 dilutions with Odyssey blocking buffer. Goat anti-rabbit IRDye® 800 CW (LI-COR®) secondary antibody was used at 1:15,000 dilution in Odyssey blocking buffer and detection was by ODYSSEY imaging system (LI-COR®).

#### Immunolocalisation

Freshly perfused *S. mansoni* worm pairs were fixed in 75% acetone and 25% ethanol at RT for 5 min. OCT blocks containing 30 worm pairs each were stored at −80 °C until sectioned. Frozen sections (7 μm thick) were cut onto SuperFrost® glass slides (MENZEL-GLÄSER) and stored at −80 °C until needed. Sections were thawed and rehydrated before blocking with 5% goat serum, 5% FBS and 2% BSA in PBS. Sections were then incubated for 1 hour in primary antibody (against *Sm*-LAMP and *Sm*-NPC2) diluted 1:200 in the blocking buffer, washed and then secondary antibody, goat anti-rabbit Alexa Flour® 488 (Invitrogen) was added after diluting 1:100 in blocking buffer. Sections were mounted in ProLong® Gold Antifade agent with DAPI (Invitrogen) after 3 washes in PBS. Sections were protected from light until examination by fluorescence microscopy using a Leica DMIRB Inverted research microscope.

#### Vaccine trials

**Figure 8:**
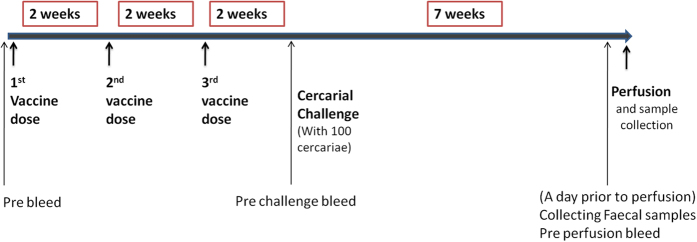
Vaccination schedule for mice. Female CBA mice were immunised with *Sm*-LAMP and *Sm*-NPC2 recombinant proteins adjuvanted with Alum-CpG. Arrows indicate the immunisation time points and those at which the samples were taken.

Groups of ten, eleven week old female CBA mice were used in blinded vaccine trials (Fig. 8). Each mouse was vaccinated with three doses of 25–30 μg of recombinant protein/PBS combined with an equal volume of Aluminium Hydroxide gel (alum) (Sigma) and 10 μg of CpG ODN1826 (InvivoGen) at two weekly intervals. The control group was given PBS and adjuvant only. The total volume of a single vaccine dose was 100 μl of which 50 μl was given subcutaneously to each inguinal region/flank. After three vaccine doses, the mice were challenged with 100 *S. mansoni* cercariae per mouse. Perfusion of the mice occurred seven weeks after cercarial challenge. In the first vaccine trial, the proteins for all three candidates were insoluble. A repeat vaccine trial was conducted using only soluble and insoluble *Sm*-LAMP.

One day prior to perfusion, grid cages were used to collect faecal samples from mice. Faecal samples were weighed and stored in 4% formalin until processed.

Blood (10 μl) was obtained from each mouse by tail bleed before the commencement of the vaccine trial and again before the cercarial challenge. Each blood sample was diluted with 90 μl of PBS. The serum was separated, aliquoted, and stored at −80 °C until required.

Mice were perfused as previously reported^14^,^65^ and the numbers of worm pairs and single males noted, and stored in 4% formalin for subsequent morphological assessment. A portion of mouse liver was stored in 4% (v/v) formalin for histological analysis of granuloma volume, while the rest was weighed and stored in dry tubes at −20 °C for egg counts. Formalin fixed mouse livers were used for the preparation of Haematoxylin and Eosin (H&E) stained sections after paraffin embedding for histological analysis of granulomas. H &E sections were scanned using the Aperio slide scanner (Aperio, Germany) and the liver granuloma density was quantified using the Aperio Imagescope v11.1.2.760 software as described earlier^66^. The small intestine was dissected, cleaned and washed in PBS and 1 cm of the middle region of the small intestine dissected and pressed between a cover slip and a glass microscope slide to make oograms^67^ and examined on the same day to calculate the percentages of mature and immature eggs present. The remainder of the intestine was weighed and stored in a dry tube at −20 °C for egg counting. Faecal eggs counts per gram of faeces were calculated using standard methods^14^,^68^. Male and female worm lengths were measured in 10 worm pairs per each group using an Olympus CKX41 inverted microscope. Worm lengths from the vaccinated groups were compared with the control group to determine the effect of vaccination on worm growth and development.

Statistical analyses of vaccine data were performed using the Mann-Whitney U test. Percentage reductions in adult worm burden, female worm burden, and hepatic, intestinal and faecal egg burdens were calculated using the formula adopted previously^69^.

Serum collected from mice in control and vaccine groups after the completion of vaccination schedule was tested for antibody titres of different immunoglobulin subtypes (IgG, IgG1, IgG2a, IgG2b and IgG3) using ELISA. Serial two fold dilutions were made from the 10× prediluted serum using blocking solution. Serum from the bleed before the first dose of vaccine was taken as the negative control. MaxiSorp® 96 well plates (Nunc) were coated with 0.5 μg of protein per well overnight at 4 °C. Plates were blocked with 3% skim milk in 0.1% PBST for one hour at 37 °C. Two hundred microlitres (200 μl) of serially diluted mouse serum from the vaccine trials (primary mouse antibodies) were added into corresponding wells. Plates were then incubated for one hour at 37 °C. After removing the primary antibody, plates were washed three times with 0.1% PBST and incubated with secondary anti mouse antibodies, IgG, IgG1, IgG2a, IgG2b and IgG3 (Invitrogen) diluted 1:2000, for 1 hour at 37 °C. SIGMA*FAST*™ OPD (*o*-phenylenediamine dihydrochloride) (Sigma) was used as the substrate and the absorption was read at 450 nm using a Bio Tek Synergy 4 plate reader.

## Additional Information

**How to cite this article**: Nawaratna, S. S. K. *et al*. Lysosome-associated membrane glycoprotein (LAMP) – preliminary study on a hidden antigen target for vaccination against schistosomiasis. *Sci. Rep*. **5**, 15069; doi: 10.1038/srep15069 (2015).

## Supplementary Material

Supplementary Information

## Figures and Tables

**Table 1:** Summary of key results for vaccine trials for *Sm*-LAMP.

Antibody end point titres	Vaccine trial 1	Vaccine trial 2	
	Insoluble LAMP	Insoluble LAMP	Soluble LAMP
**IgG**	1:32 000 (1:100)	1:128 000 (1:100)	1:256 000 (1:100)
**IgG1**	1:128 000 (1:100)	1: 32 000 (1:100)	1:256 000 (1:100)
**IgG2a**	1:64 000 (1:50)	1:128 000 (1:50)	1:64 000 (1:50)
**IgG2b**	1:128 000 (1:50)	1:32 000 (1:50)	1:128 000 (1:50)
**IgG3**	NR	NR	1:64 000
**Worm counts**			
**Females -% reduction**	20.93 (p = 0.04)	35.7 (p = 0.019)	16 (NS)
**Total -% reduction**	16.21 (p = 0.04)	25 (p = 0.01)	8.7 (NS)
**Egg counts**			
**Liver -% reduction**	20 (p = 0.021)	38 (p = 0.015)	13 (NS)
**Intestine -% reduction**	21 (p = 0.011)	49 (NS)	30 (NS)
**Faecal -% reduction**	51.9 (p = 0.001)	60 (p = 0.009)	54 (p = 0.035)
**Faecal egg reduction per female**	56.2 (NS)	22.3 (NS)	58.6 (NS)

The antibody titres in brackets show the control values.

NR = No response.

NS = No statistical significance.

## References

[b1] King C. H., Dickman K. & Tisch D. J. Reassessment of the cost of chronic helmintic infection: a meta-analysis of disability-related outcomes in endemic schistosomiasis. Lancet 365, 1561–9 (2005). 15866310 10.1016/S0140-6736(05)66457-4

[b2] Guisse F. . Therapeutic evaluation of two different dose regimens of praziquantel in a recent *Schistosoma mansoni* focus in Northern Senegal. Am J Trop Med Hyg 56, 511–4 (1997). 9180600 10.4269/ajtmh.1997.56.511

[b3] Gryseels B. . Are poor responses to praziquantel for the treatment of Schistosoma mansoni infections in Senegal due to resistance? An overview of the evidence. Tropical medicine & international health 6, 864–73 (2001). 11703840 10.1046/j.1365-3156.2001.00811.x

[b4] Stelma F. F. . Efficacy and side effects of praziquantel in an epidemic focus of *Schistosoma mansoni*. Am J Trop Med Hyg 53, 167–70 (1995). 7677219 10.4269/ajtmh.1995.53.167

[b5] Ismail M. . Resistance to praziquantel: direct evidence from *Schistosoma mansoni* isolated from Egyptian villagers. Am J Trop Med Hyg 60, 932–5 (1999). 10403323 10.4269/ajtmh.1999.60.932

[b6] Day T. A., Bennett J. L. & Pax R. A. Praziquantel: The enigmatic antiparasitic. Parasitol Today 8, 342–4 (1992). 15463532 10.1016/0169-4758(92)90070-i

[b7] Smithers S. R. Immunizing effect of irradiated cercariae of Schistosoma mansoni in rhesus monkeys. Nature 194, 1146–7 (1962). 13914469 10.1038/1941146a0

[b8] Hsu S. Y., Hsu H. F. & Osborn J. W. Immunization of rhesus monkeys against schistosome infection by cercariae exposed to high doses of x-radiation. Proc Soc Exp Biol Med. 131, 1146–9 (1969). 4980828

[b9] Bickle Q. D. Radiation-attenuated schistosome vaccination—a brief historical perspective. Parasitology 136, 1621–32 (2009). 19327194 10.1017/S0031182009005848

[b10] Da’dara A. A. . DNA-based vaccines protect against zoonotic schistosomiasis in water buffalo. Vaccine 26, 3617–25 (2008). 18524429 10.1016/j.vaccine.2008.04.080 PMC2567122

[b11] Ahmad G., Torben W., Zhang W., Wyatt M. & Siddiqui A. A. Sm-p80-based DNA vaccine formulation induces potent protective immunity against *Schistosoma mansoni*. Parasite Immunol 31, 156–61 (2009). 19222788 10.1111/j.1365-3024.2008.01091.x PMC2786212

[b12] Bergquist N. R. Schistosomiasis vaccine development: approaches and prospects. Mem Inst Oswaldo Cruz 90, 221–7 (1995). 8531662 10.1590/s0074-02761995000200017

[b13] Fonseca C. T., Oliveira S. C. & Alves C. C. Eliminating Schistosomes through Vaccination: What are the Best Immune Weapons? Frontiers Immunol 6, 95 (2015). 10.3389/fimmu.2015.00095 PMC4353369 25806033

[b14] Tran M. H. . Tetraspanins on the surface of *Schistosoma mansoni* are protective antigens against schistosomiasis. Nat Med 12, 835–40 (2006). 16783371 10.1038/nm1430

[b15] Cardoso F. C., Pacifico R. N., Mortara R. A. & Oliveira S. C. Human antibody responses of patients living in endemic areas for schistosomiasis to the tegumental protein Sm29 identified through genomic studies. Clin Exp Immunol 144, 382–91 (2006). 16734606 10.1111/j.1365-2249.2006.03081.x PMC1941986

[b16] Cardoso F. C. . Schistosoma mansoni tegument protein Sm29 is able to induce a Th1-type of immune response and protection against parasite infection. PLoS Negl Trop Dis 2, e308 (2008). 18827884 10.1371/journal.pntd.0000308 PMC2553283

[b17] Brindley P. J. . Proteolytic degradation of host hemoglobin by schistosomes. Mol Biochem Parasitol 89, 1–9 (1997). 9297696 10.1016/s0166-6851(97)00098-4

[b18] Caffrey P. New start and finish for complex polyketide biosynthesis. Chem Biol 11, 155–7 (2004). 15123275 10.1016/j.chembiol.2004.02.002

[b19] Halton D. W. Nutritional adaptations to parasitism within the platyhelminthes. Int J Parasitol 27, 693–704 (1997). 9229252 10.1016/s0020-7519(97)00011-8

[b20] Skelly P. J., Da’dara A. A., Li X. H., Castro-Borges W. & Wilson R. A. Schistosome feeding and regurgitation. PLoS Path 10, e1004246 (2014). 10.1371/journal.ppat.1004246 PMC4133383 25121497

[b21] Wilson R. A. The saga of schistosome migration and attrition. Parasitology 136, 1581–92 (2009). 19265564 10.1017/S0031182009005708

[b22] Hall S. L. . Insights into blood feeding by schistosomes from a proteomic analysis of worm vomitus. Mol Biochem Parasitol 179, 18–29 (2011). 21605600 10.1016/j.molbiopara.2011.05.002

[b23] Munn E. A. Rational design of nematode vaccines: hidden antigens. Int J Parasitol 27, 359–66 (1997). 9184927 10.1016/s0020-7519(97)00003-9

[b24] Knox D. P. . The nature and prospects for gut membrane proteins as vaccine candidates for *Haemonchus contortus* and other ruminant trichostrongyloids. Int J Parasitol 33, 1129–37 (2003). 13678629 10.1016/s0020-7519(03)00167-x

[b25] Newton S. E. & Munn E. A. The development of vaccines against gastrointestinal nematode parasites, particularly *Haemonchus contortus*. Parasitol Today 15, 116–22 (1999). 10322325 10.1016/s0169-4758(99)01399-x

[b26] Willadsen P., Bird P., Cobon G. S. & Hungerford J. Commercialisation of a recombinant vaccine against *Boophilus microplus*. Parasitology 110 **Suppl**, S43–50 (1995). 7784128 10.1017/s0031182000001487

[b27] Nawaratna S. S., McManus D. P., Moertel L., Gobert G. N. & Jones M. K. Gene Atlasing of digestive and reproductive tissues in Schistosoma mansoni. PLoS Negl Trop Dis 5, e1043 (2011). 21541360 10.1371/journal.pntd.0001043 PMC3082511

[b28] Nawaratna S. S., McManus D. P., Moertel L., Gobert G. N. & Jones M. K. Gene Atlasing of digestive and reproductive tissues in Schistosoma mansoni. PLoS Negl Trop Dis 5, e1043 (2011). 21541360 10.1371/journal.pntd.0001043 PMC3082511

[b29] Gobert G. N. . Tissue specific profiling of females of Schistosoma japonicum by integrated laser microdissection microscopy and microarray analysis. PLoS Negl Trop Dis 3, e469 (2009). 19564906 10.1371/journal.pntd.0000469 PMC2696939

[b30] Jones D. T. Protein secondary structure prediction based on position-specific scoring matrices. J Mol Biol 292, 195–202 (1999). 10493868 10.1006/jmbi.1999.3091

[b31] Albertti L. A., Macedo A. M., Chiari E., Andrews N. W. & Andrade L. O. Role of host lysosomal associated membrane protein (LAMP) in *Trypanosoma cruzi* invasion and intracellular development. Microbes Infect 12, 784–789 (2010). 20561595 10.1016/j.micinf.2010.05.015 PMC2934878

[b32] Wilke S., Krausze J. & Bussow K. Crystal structure of the conserved domain of the DC lysosomal associated membrane protein: implications for the lysosomal glycocalyx. BMC Biology 10, 62 (2012). 22809326 10.1186/1741-7007-10-62 PMC3409847

[b33] Eskelinen E. L. . Disturbed cholesterol traffic but normal proteolytic function in LAMP-1/LAMP-2 double-deficient fibroblasts. Mol Biol Cell 15, 3132–45 (2004). 15121881 10.1091/mbc.E04-02-0103 PMC452571

[b34] Schneede A. . Role for LAMP-2 in endosomal cholesterol transport. J Cell Mol Med 15, 280–95 (2011). 19929948 10.1111/j.1582-4934.2009.00973.x PMC3822795

[b35] Kostich M., Fire A. & Fambrough D. M. Identification and molecular-genetic characterization of a LAMP/CD68-like protein from *Caenorhabditis elegans*. J Cell Sci 113 2595–606 (2000). 10862717 10.1242/jcs.113.14.2595

[b36] de Saint-Vis B. . A novel lysosome-associated membrane glycoprotein, DC-LAMP, induced upon DC maturation, is transiently expressed in MHC class II compartment. Immunity 9, 325–36 (1998). 9768752 10.1016/s1074-7613(00)80615-9

[b37] Braulke T. & Bonifacino J. S. Sorting of lysosomal proteins. Biochim Biophys Acta 1793, 605–14 (2009). 19046998 10.1016/j.bbamcr.2008.10.016

[b38] Zhang Z. . MicroRNAs: potential regulators involved in human anencephaly. Int J Biochem Cell Biol 42, 367–74 (2010). 19962448 10.1016/j.biocel.2009.11.023

[b39] Zhang H. . Full length amelogenin binds to cell surface LAMP-1 on tooth root/periodontium associated cells. Arch Oral Biol 55, 417–25 (2010). 20382373 10.1016/j.archoralbio.2010.03.009 PMC2886511

[b40] Cass C. L. . Proteomic analysis of *Schistosoma mansoni* egg secretions. Mol Biochem Parasitol 155, 84–93 (2007). 17644200 10.1016/j.molbiopara.2007.06.002 PMC2077830

[b41] Infante R. E. . NPC2 facilitates bidirectional transfer of cholesterol between NPC1 and lipid bilayers, a step in cholesterol egress from lysosomes. Proc Natl Acad Sci USA 105, 15287–92 (2008). 18772377 10.1073/pnas.0807328105 PMC2563079

[b42] Storch J. & Xu Z. Niemann-Pick C2 (NPC2) and intracellular cholesterol trafficking. Biochim Biophys Acta 1791, 671–8 (2009). 19232397 10.1016/j.bbalip.2009.02.001 PMC4281484

[b43] Busso D., Onate-Alvarado M. J., Balboa E., Zanlungo S. & Moreno R. D. Female infertility due to anovulation and defective steroidogenesis in NPC2 deficient mice. Mol Cell Endocrinol 315, 299–307 (2010). 19883728 10.1016/j.mce.2009.10.011

[b44] Liscum L. A role for NPC1 and NPC2 in intestinal cholesterol absorption—the hypothesis gutted. Biochem J 408, e1–3 (2007). 17956226 10.1042/BJ20071340 PMC2049072

[b45] Dixit S. S., Sleat D. E., Stock A. M. & Lobel P. Do mammalian NPC1 and NPC2 play a role in intestinal cholesterol absorption? Biochem J 408, 1–5 (2007). 17880278 10.1042/BJ20071167 PMC2049080

[b46] Bansal D., Bhatti H. S. & Sehgal R. Role of cholesterol in parasitic infections. Lipids Health Dis 4, 10 (2005). 15882457 10.1186/1476-511X-4-10 PMC1142336

[b47] Berriman M. . The genome of the blood fluke *Schistosoma mansoni*. Nature 460, 352–8 (2009). 19606141 10.1038/nature08160 PMC2756445

[b48] Doenhoff M. J., Stanley R. G., Griffiths K. & Jackson C. L. An anti-atherogenic effect of *Schistosoma mansoni* infections in mice associated with a parasite-induced lowering of blood total cholesterol. Parasitology 125, 415–21 (2002). 12458825 10.1017/s0031182002002275

[b49] La Flamme A. C. . Chronic exposure to schistosome eggs reduces serum cholesterol but has no effect on atherosclerotic lesion development. Parasite Immunol 29, 259–66 (2007). 17430549 10.1111/j.1365-3024.2007.00942.x

[b50] Verity C. K., McManus D. P. & Brindley P. J. Vaccine efficacy of recombinant cathepsin D aspartic protease from *Schistosoma japonicum*. Parasite Immunol 23, 153–62 (2001). 11240906 10.1046/j.1365-3024.2001.00369.x

[b51] Wilson R. A. & Coulson P. S. Schistosome vaccines: a critical appraisal. Mem Inst Oswaldo Cruz 101 **Suppl 1**, 13–20 (2006). 17308743 10.1590/s0074-02762006000900004

[b52] Loukas A., Tran M. & Pearson M. S. Schistosome membrane proteins as vaccines. Int J Parasitol 37, 257–63 (2007). 17222846 10.1016/j.ijpara.2006.12.001

[b53] Hofmeister Y. . Human IgG subclasses: *in vitro* neutralization of and *in vivo* protection against West Nile virus. J Virol 85, 1896–9 (2011). 21123389 10.1128/JVI.02155-10 PMC3028917

[b54] Huber V. C. . Distinct contributions of vaccine-induced immunoglobulin G1 (IgG1) and IgG2a antibodies to protective immunity against influenza. Clin Vacc Immunol CVI 13, 981–90 (2006). 10.1128/CVI.00156-06 PMC1563571 16960108

[b55] Pearce E. J. & MacDonald A. S. The immunobiology of schistosomiasis. Nat Rev Immunol 2, 499–511 (2002). 12094224 10.1038/nri843

[b56] Nawaratna S. S. . Transcriptional profiling of the oesophageal gland region of male worms of Schistosoma mansoni. Mol Biochem Parasitol 196, 82–89 (2014). 25149559 10.1016/j.molbiopara.2014.08.002

[b57] Figueiredo B. C.-P. . Kicking in the guts: Schistosoma mansoni digestive tract proteins are potential candidates for vaccine development. Frontiers in Immunology 6 (2015), 10.3389/fimmu.2015.00022. PMC4309203 25674091

[b58] Martins V. P. . Sm10.3, a member of the micro-exon gene 4 (MEG-4) family, induces erythrocyte agglutination *in vitro* and partially protects vaccinated mice against Schistosoma mansoni infection. PLoS Negl Trop Dis 8, e2750 (2014). 24651069 10.1371/journal.pntd.0002750 PMC3961193

[b59] Conesa A. . Blast2GO: a universal tool for annotation, visualization and analysis in functional genomics research. Bioinformatics 21, 3674–6 (2005). 16081474 10.1093/bioinformatics/bti610

[b60] Larkin M. A. . Clustal W and Clustal X version 2.0. Bioinformatics 23, 2947–8 (2007). 17846036 10.1093/bioinformatics/btm404

[b61] Moertel L., Gobert G. N. & McManus D. P. Comparative real-time PCR and enzyme analysis of selected gender-associated molecules in *Schistosoma japonicum*. Parasitology 135, 575–83 (2008). 18294425 10.1017/S0031182008004174 PMC2754247

[b62] Gobert G. N. . Transcriptional changes in Schistosoma mansoni during early schistosomula development and in the presence of erythrocytes. PLoS Negl Trop Dis 4, e600 (2010). 20161728 10.1371/journal.pntd.0000600 PMC2817720

[b63] Moertel L. . Oligonucleotide microarray analysis of strain- and gender-associated gene expression in the human blood fluke, Schistosoma japonicum. Mol Cell Probes 20, 280–9 (2006). 16647836 10.1016/j.mcp.2006.02.002

[b64] Hofmann A. ACDP - a Java application for data processing and analysis of protein circular dichroism spectra. J Appl Cryst. 42, 137–139 (2009).

[b65] Chuah C. . Spatial and temporal transcriptomics of Schistosoma japonicum-induced hepatic granuloma formation reveals novel roles for neutrophils. J Leuk Biol 94, 353–65 (2013). 10.1189/jlb.1212653 23709687

[b66] You H. . Suppression of the Insulin Receptors in Adult Schistosoma japonicum Impacts on Parasite Growth and Development: Further Evidence of Vaccine Potential. PLoS Negl Trop Dis 9, e0003730 (2015). 25961574 10.1371/journal.pntd.0003730 PMC4427307

[b67] Pellegrino J., Oliveira C. A., Faria J. & Cunha A. S. New approach to the screening of drugs in experimental schistosomiasis mansoni in mice. Am J Trop Med Hyg 11, 201–15 (1962). 14484966 10.4269/ajtmh.1962.11.201

[b68] You H. . The insulin receptor is a transmission blocking veterinary vaccine target for zoonotic *Schistosoma japonicum*. Int J Parasitol 42, 801–7 (2012). 22771861 10.1016/j.ijpara.2012.06.002

[b69] Xu X. . A *Schistosoma japonicum* chimeric protein with a novel adjuvant induced a polarized Th1 immune response and protection against liver egg burdens. BMC Infect Dis 9, 54 (2009). 19419545 10.1186/1471-2334-9-54 PMC2685138

