# Supplementary material for: Lysosome-associated membrane glycoprotein (LAMP) – preliminary study on a hidden antigen target for vaccination against schistosomiasis
Source: Sci Rep. 2015 Oct 16;5:15069. doi: 10.1038/srep15069 (PMC4607944; doi:10.1038/srep15069)
Supplement: Supplementary Information [file srep15069-s1.doc]

**Lysosome-associated membrane glycoprotein (LAMP) – preliminary study on a hidden antigen target for vaccination against schistosomiasis**

Sujeevi S.K. Nawaratna^1,2^, Geoffrey N. Gobert^2^, Charlene Willis^2^, Jason Mulvenna^2^, Andreas

Hofmann^3,4^, Donald P McManus2 & Malcolm K Jones^1,2^

**Supplementary Table 1.** List of oligonucleotides used for real-time PCR

|  | Forward primer | Reverse primer |
| --- | --- | --- |
| Real-time PCR Primers | | |
| LAMP | 5’ACAGCAACAACAACCGATGA3’ | 5’CTGCCGTTGTTTCTTTCGTT3’ |
| NPC2 | 5’TGATTGTTCTGTGGCTTGCT3’ | 5’ACATGGCGTTACAGTTAGTGAA3’ |

**Supplimentary Figure 1.**

LAMP2_HUMAN MVCFRLFPVPGSGLVLVCLVLGAVRSYALELNLTDSENATCLYAKWQMNFTVRYETTN-K 59

LAMP2_MOUSE ---MCLSPVKGAKLILIFLFLGAVQSNALIVNLTDSK-GTCLYAEWEMNFTITYETTN-Q 55

LAMP1_HUMAN MAAPGSARRPLLLLLLLLLLGLMHCASAAMFMVKNGNGTACIMANFSAAFSVNYDTKSGP 60

LAMP1_MOUSE MAAPG-ARRP---LLLLLLAGLAHGAS-ALFEVKN-NGTTCIMASFSASFLTTYETANGS 54

Smp_073400 ------------------------------------------------------------

Sjp_0002430 ------------------------------------------------------------ Smp_032520 ------------------------------------------------------------ Smp_039620 ------------------------------------------------------------ LMP1_C. ------------------------------------------------------------

Sm-LAMP --------------------------------------------------MLPGSSVYIE 10

LAMP2_HUMAN TYKTVTISDHGTVTYNGSICG-DDQNGPKIAVQFGPGFSWIANFTKAASTYSIDSVSFSY 118

LAMP2_MOUSE TNKTITIAVPDKATHDGSSCG-DDRNSAKIMIQFGFAVSWAVNFTKEASHYSIHDIVLSY 114

LAMP1_HUMAN KNMTFDLPSDATVVLNRSSCGKENTSDPSLVIAFGRGHTLTLNFTRNATRYSVQLMSFVY 120

LAMP1_MOUSE QIVNISLPASAEVLKNGSSCGKENVSDPSLTITFGRGYLLTLNFTKNTTRYSVQHMYFTY 114

Smp_073400 ------------------------------------------------------------

Sjp_0002430 ------------------------------------------------------------

Smp_032520 ------------------------------------------------------------ Smp_039620 ------------------------------------------------------------ LMP1_C. ------------------------------------------------------------

Sm-LAMP LLFLVLLSHSSSDVYGFTDADGYKSSSQSSETEETEVTDYSDDIIFPVINVSYDNMTANG 70

| LAMP2_HUMAN | NTGDNTTFPDAEDKGILTVDELLAIRIPLNDLFRCNSLSTLEKNDVVQHYWDVLVQAFVQ | 178 |
| --- | --- | --- |
| LAMP2_MOUSE | NTSDSTVFPGAVAKGVHTVKNPENFKVPLDVIFKCNSVLTYNLTPVVQKYWGIHLQAFVQ | 174 |
| LAMP1_HUMAN | NLSDTHLFPNASSKEIKTVESITDIRADIDKKYRCVSGTQVHMNNVTVTLHDATIQAYLS | 180 |
| LAMP1_MOUSE | NLSDTEHFPNAISKEIYTMDSTTDIKADINKAYRCVSDIRVYMKNVTVVLRDATIQAYLS | 174 |
| Smp_073400 | -------------------MLLLDMRSTLLCFILCISSSVVLARKEINELATAKPSTFNV | 41 |
| Sjp_0002430 | ------------------------------------------------------------ |  |
| Smp_032520 | -----------------------MFVALSALIVACLDQIVCYSVDPVTSPATFRIGSN-- | 35 |
| Smp_039620 | ----------------------------MQQIKGSKDNITSYLN---------------- | 16 |
| LMP1_C. | ------------------------MLKSFVILFAFLASASATASHYYVTNNNTGLTCIIL | 36 |
| Sm-LAMP | TTVPYLPTATTTDEPNSTIRESTTTVLPNTTDHSHDSTIATETTSTTETATTRGMTTAGV | 130 |

| LAMP2_HUMAN | NGTVSTNEFLCDKDKT-STVAPTIHTTVPSPTT--------TPTPKEKPEAGTYSVNNGN | 229 |
| --- | --- | --- |
| LAMP2_MOUSE | NGTVSKNEQVCEEDQTPTTVAPIIHTTAPSTTTTLTPTSTPTPTPTPTPTVGNYSIRNGN | 234 |
| LAMP1_HUMAN | NSSFSRGETRCEQDRPSPTTAP---PAPPSPSP---------SPVPKSPSVDKYNVSGTN | 228 |
| LAMP1_MOUSE | SGNFSKEETHCTQDGPSPTTG------PPSPSP---------PLVPTNPTVSKYNVTGNN | 219 |
| Smp_073400 | DGCLLLSA-------------------------------------IMKIHISKSDPVKQS | 64 |
| Sjp_0002430 | ----------------------------------------------MRDHIR-------- | 6 |
| Smp_032520 | DSSILLQG-------------------------------------YINITLAYTKVQKAP | 58 |
| Smp_039620 | ------------------------------------------------------------ |  |
| LMP1_C. | DGDFQFNLVFNEKN------------------------------TTEKFTVTFNETVSVE | 66 |
| Sm-LAMP | TTTKETTAG------------------------------------VTTTEVTTAGVTTTE | 154 |

| LAMP2_HUMAN | DTCLLATMGLQLNITQDK-----VASVININPNTTHSTGSCRSHTALLRLNSSTIKYLDF | 284 |
| --- | --- | --- |
| LAMP2_MOUSE | TTCLLATMGLQLNITEEK-----VPFIFNINPATTNFTGSCQPQSAQLRLNNSQIKYLDF | 289 |
| LAMP1_HUMAN | GTCLLASMGLQLNLTYERKDNTTVTRLLNINPNKTSASGSCGAHLVTLELHSEGTTVLLF | 288 |
| LAMP1_MOUSE | GTCLLASMALQLNITYLKKDNKTVTRAFNISPNDTS-SGSCGINLVTLKVENK-NRALEL | 277 |
| Smp_073400 | ESVFTTYINSTN------------AKSINSSGECMNSTSREYNLLNLCWKPNGSEGNWNI | 112 |
| Sjp_0002430 | --------------------------ALQD------------------------------ | 10 |
| Smp_032520 | -------VVVSI------------DSATQGKFQVSGVLGNEIEGLNLSWKPDQSNSTWNL | 99 |
| Smp_039620 | --------------------------STVKNIDFFGYCDSNLNSLTVKWADPNQKNPWLL | 50 |
| LMP1_C. | GDCN--------------------GVRNNQSVQTLNIKFNPEGQSARYAKEWELDIVFGS | 106 |
| Sm-LAMP | VITKEMETTAVMTTTDMITTVVPIPSVHKFSLKSGNSYCFLFQSDIMFQVFYNSHSKLVK | 214 |

| LAMP2_HUMAN | VFAVKNEN-RFYLKEVNISMYLVNG--SVFSIANNNLSYWDAPLGSSYMCNKEQTVSVSG | 341 |
| --- | --- | --- |
| LAMP2_MOUSE | IFAVKNEK-RFYLKEVNVYMYLANG--SAFNISNKNLSFWDAPLGSSYMCNKEQVLSVSR | 346 |
| LAMP1_HUMAN | QFGMNASSSRFFLQGIQLNTILPDARDPAFKAANGSLRALQATVGNSYKCNAEEHVRVTK | 348 |
| LAMP1_MOUSE | QFGMNASSSLFFLQGVRLNMTLPDALVPTFSISNHSLKALQATVGNSYKCNTEEHIFVSK | 337 |
| Smp_073400 | SFNFSETYPGYYGLTSVYLLYWLDK--LGPHNASTDKSLFSCAIGTSFVCLSEQTYELKD | 170 |
| Sjp_0002430 | ----------------------------GPLNASTDKSLFSCAIGSSFICVSEQTFELKD | 42 |
| Smp_032520 | TFRFAKSENSYNLSEISFVYELLDL--PGLRYASNNHSLFSAAIGSYYSCQAEQNLVLNH | 157 |
| Smp_039620 | TFNFKLYANDFYAFDSITFFYRLND---EQIYASSDDEVFSIQKDQYYNCTKALKIELRP | 107 |
| LMP1_C. | SSNVAFEIIDYTLTTQRTDLVPYFG---KFVRDANAAGDVTATQTNAYKCSTAKLGLVGG | 163 |
| Sm-LAMP | SYKFTLSNATINDNESVCSEHYTKLSLTFIPYGRNEEHKWTLVLLFNRMEPNKTSNDATN | 274 |

| LAMP2_HUMAN | ------AFQINTFDLRVQPFNVT----QGKYSTAQDCSADDD-NFLVPIAVGAALAGVLI | 390 |
| --- | --- | --- |
| LAMP2_MOUSE | ------AFQINTFNLKVQPFNVT----KGQYSTAQDCSADED-NFLVPIAVGAALGGVLI | 395 |
| LAMP1_HUMAN | ------AFSVNIFKVWVQAFKVE----GGQFGSVEECLLDEN-SMLIPIAVGGALAGLVL | 397 |
| LAMP1_MOUSE | ------MLSLNVFSVQVQAFKVD----SDRFGSVEECVQDGN-NMLIPIAVGGALAGLVL | 386 |
| Smp_073400 | KLSNSTNIRLTFSEFQVEAFRNNDISNNTFTGPTSSCAADYVPTKVIPIVVGVLLVVMIA | 230 |
| Sjp_0002430 | KSSNSTNIRFTFSQFQVEAFRSNSTDNNTFTGPTTSCSADYVPTKVIPIVVGVLLVIMIA | 102 |
| Smp_032520 | FAPNPVTVNLTFSQLKVQAFRHG--LEPEFNGIMVQCSLDYHLDRVVPIVIGISLAVMII | 215 |
| Smp_039620 | SDRNYSTVRLIFNSIEVEAFRES--SGTSYVGKESHCSKDDK-ENLTYIIVSISIFTMVV | 164 |
| LMP1_C. | -------STIDIKTSNVIAFAQMNGTVFPTDQVYEVCYLDARTNEVVPIVVGACLAALVV | 216 |
| Sm-LAMP | ------VSSIYTLDSITLTYYMDKNLFPDSLTPETLCDSDVNVNNMVPIGVGIALIVCIV  . . .: * * : * :. : : | 328 |

LAMP2_HUMAN LVLLAYFIGLKHHH--------AGYEQF- 410

| LAMP2_MOUSE | LVLLAYFIGLKRHH--------TGYEQF- | 415 |
| --- | --- | --- |
| LAMP1_HUMAN | IVLIAYLVGRKRSH--------AGYQTI- | 417 |
| LAMP1_MOUSE | IVLIAYLIGRKRSH--------AGYQTI- | 406 |
| Smp_073400 | AALIAFIISSRRRQ--------IGYEEI- | 250 |
| Sjp_0002430 | AALIAFIISSRRRQ--------VGYEEI- | 122 |
| Smp_032520 | VALIAFIITSRRNRNISGNGVSGGYQQI- | 243 |
| Smp_039620 | VMIFVLCLSNDETQ-------VVGVDHYR | 186 |
| LMP1_C. | VVLVGYLIGRARAK-------RQGYASV- | 237 |
| Sm-LAMP | VAITVFIVFNKRNR--------RSYTTL- | 348 |
|  | : : . : . |  |

CLUSTAL 2.1 Multiple Sequence Alignments

Sequence type explicitly set to Protein

Sequence format is Pearson

Sequence 1: Sm-LAMP 348 aa (Smp_162770) Sequence 2: Smp_032520 243 aa

Sequence 3: Smp_039620 186 aa Sequence 4: Smp_073400 250 aa Sequence 5: Sjp_0002430 122 aa Sequence 6: LAMP2_HUMAN 410 aa Sequence 7: LAMP1_HUMAN 417 aa Sequence 8: LAMP2_MOUSE 415 aa Sequence 9: LAMP1_MOUSE 406 aa

Sequence 10: LMP1_C. 237 aa (*C.elegance*)

Start of Pairwise alignments

Sequences (1:2) Aligned. Score: 15.2263

Sequences (1:3) Aligned. Score: 12.3656

Sequences (1:4) Aligned. Score: 14.4

Sequences (1:5) Aligned. Score: 20.4918

Sequences (1:6) Aligned. Score: 14.3678

Sequences (1:7) Aligned. Score: 12.931

Sequences (1:8) Aligned. Score: 15.2299

Sequences (1:9) Aligned. Score: 14.0805

Sequences (1:10) Aligned. Score: 15.6118

Sequences (2:3) Aligned. Score: 20.4301

Sequences (2:4) Aligned. Score: 27.572

Sequences (2:5) Aligned. Score: 36.8852

Sequences (2:6) Aligned. Score: 13.5802

Sequences (2:7) Aligned. Score: 16.4609

Sequences (2:8) Aligned. Score: 15.2263

Sequences (2:9) Aligned. Score: 14.8148

Sequences (2:10) Aligned. Score: 17.7215

Sequences (3:4) Aligned. Score: 19.3548

Sequences (3:5) Aligned. Score: 20.4918

Sequences (3:6) Aligned. Score: 12.9032

Sequences (3:7) Aligned. Score: 12.9032

Sequences (3:8) Aligned. Score: 13.4409

Sequences (3:9) Aligned. Score: 12.9032

Sequences (3:10) Aligned. Score: 13.4409

Sequences (4:5) Aligned. Score: 78.6885

Sequences (4:6) Aligned. Score: 14.4

Sequences (4:7) Aligned. Score: 12.8

Sequences (4:8) Aligned. Score: 14.4

Sequences (4:9) Aligned. Score: 15.6

Sequences (4:10) Aligned. Score: 14.7679

Sequences (5:6) Aligned. Score: 18.0328

Sequences (5:7) Aligned. Score: 19.6721

Sequences (5:8) Aligned. Score: 18.0328

Sequences (5:9) Aligned. Score: 20.4918

Sequences (5:10) Aligned. Score: 17.2131

Sequences (6:7) Aligned. Score: 30.7317

Sequences (6:8) Aligned. Score: 63.4146

Sequences (6:9) Aligned. Score: 29.803

Sequences (6:10) Aligned. Score: 15.6118

Sequences (7:8) Aligned. Score: 28.1928

Sequences (7:9) Aligned. Score: 64.2857

Sequences (7:10) Aligned. Score: 15.6118

Sequences (8:9) Aligned. Score: 28.0788

Sequences (8:10) Aligned. Score: 15.1899

Sequences (9:10) Aligned. Score: 16.8776

**Supplimentary Figure 1.** Alignment of human, mouse, C elegance and *Schistosoma* LAMP protein sequences using the ClustalW multiple sequence alignment method. Alignment scores between sequences are given below the figure. Transmembrane region is indicated by the underlying red bar. Lysosomal targeting signal in the cytosolic tail is highlighted in blue. Smp_073400, Smp_032520 and Smp_039620 are the other *Sm-* LAMPs identified in gene DB.

Supplimen tary Figure 2.


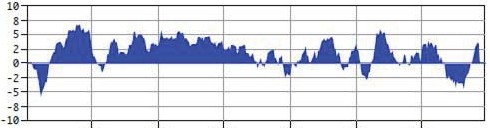
**Parker antigenicity: Window = 1 1**


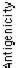


50 100 150 200 250 300


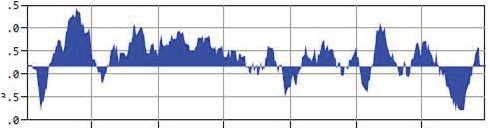
5

£ 5

*u*

· 4

;:

u

=.., 4

**Protrusion Index antigenicity: Window = 11**

3

0.


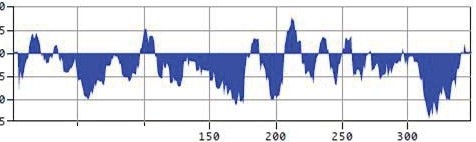


0

*z.*

·u

-0

·.c,,:.

50 100 150 200 250 300

Welling ant genicity: Window= 11

:;:; -0

c

..: -e.

-0.

1


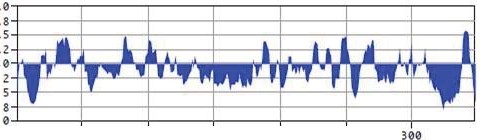


*z·u.* 0

0

**Antigenic Index: W1ndow • *7***

0

c

0

-0.

..: -0.

-0.

-1.

50 150 200 250

Supplementary Figure 2. Antigenicity ofSm-LAMP predicted using MacVector™ 8.0 software

(http://www.m acvector.com)

**Supporting information captions**

**Supplementary Table 1. List of oligonucleotides used for real-time PCR**

**Supplementary Figure** 1. Alignment of human, mouse, *C. elegance* and Schistosoma LAMP protein sequences using the ClustalW multiple sequence alignment method. Alignment scores between sequences are given below the figure. Transmembrane region is indicated by the underlying red bar. Lysosomal targeting signal in the cytosolic tail is highlighted in blue.

**Supplementary Figure 2.** Antigenicity of Sm-LAMP predicted using MacVector™ 8.0 software ([http://www.macvector.com](http://www.macvector.com/))
